# Chemical Neural Networks Inside Synthetic Cells? A Proposal for Their Realization and Modeling

**DOI:** 10.3389/fbioe.2022.927110

**Published:** 2022-06-06

**Authors:** Pier Luigi Gentili, Pasquale Stano

**Affiliations:** ^1^ Department of Chemistry, Biology and Biotechnology, Università Degli Studi di Perugia, Perugia, Italy; ^2^ Department of Biological and Environmental Sciences and Technologies (DiSTeBA), University of Salento, Lecce, Italy

**Keywords:** artificial cells, chemical AI, embodied AI, fuzzy logic, neural networks, synthetic biology, synthetic cells

## A Synthetic Biology Platform for Embodied Chemical AI

The exciting sci-tech arena of synthetic biology (SB) provides concepts, tools, and approaches for fundamental revolutions in basic and applied research. SB plays a key role when it is conceived as one of the branches of the “sciences of the artificial” (Cordeschi, 2002; Damiano et al., 2011), together with artificial intelligence (AI) and robotics. In particular, SB contributes to the wetware approaches, which are complementary to the software and hardware ones that characterize the other two most well-known branches. In this perspective, SB offers the unique opportunity of devising novel chemical versions of AI, whose main feature is *embodiment*, i.e., forms, systems, networks that *compute* through physical interactions (not based on the abstract representations typical of AI), and that potentially display autonomous adaptive/plastic dynamics (in contrast to mechanical robots).

SB, then, can be seen as an experimental platform for unconventional computing, based on (bio)chemicals, organized structures, and reactions. Operations, even when interpreted by observers in terms of logical representations, actually lie in the material domain. As such, operations (reactions, interactions, synthesis and degradation of the operators) and the operators themselves (the molecules performing or subjected to the operations) are truly interwoven, and definitely cancel the distinction between hardware and software, typical of non-chemical machines.

In this opinion paper, we aim at sketching a possible implementation of embodied, chemical AI by means of SB tools. In particular, we will focus on bottom-up approaches and on the so-called synthetic (or artificial) cells (SCs or ACs) (Luisi 2002; Salehi-Reyhan et al., 2017; Göpfrich et al., 2018; Guindani et al., 2022), Figure 1A. In the past few years, indeed, the worldwide community of SC practitioners has generated a very relevant momentum, promoted by the onset of numerous consortia and projects (Schwille et al., 2018; Frischmon et al., 2021). The question we would like to deal with is the following: is it possible to devise minimal forms of perceptive chemical AI in SCs? Because of its widespread relevance since the beginning of AI, the system we look at is a *chemical perceptron* (a *chemical neural network*), and we will discuss its possible implementation inside SCs.

**FIGURE 1:**
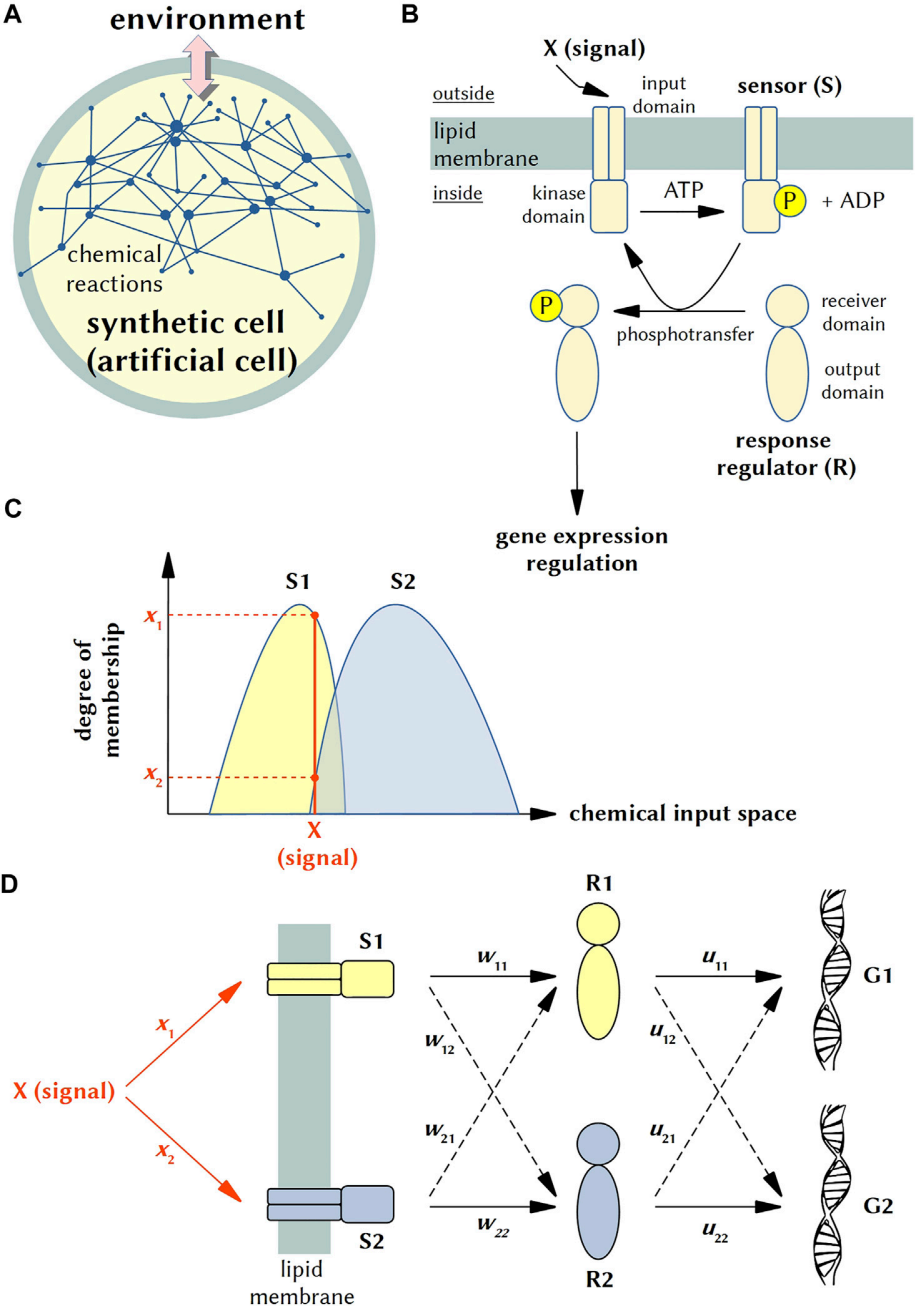
Phospho-neural network inside SCs and fuzzy logic modeling. **(A)** Synthetic (or artificial) cells (SCs) can be considered cell-like chemical systems designed and constructed in order to model some aspects of cellular behavior, such as gene expression, morphological transformations, biochemical energy production, nucleic acid duplication, signalling, cell-cell communication, etc., At this aim, a proper chemical/biochemical network (which can include membrane components) is realized within microcompartments such as lipid vesicles (liposomes), polymeric vesicles, fatty acid vesicles, coacervates, water-in-oil droplets and alike. Importantly, synthetic cells can interact with the external world (the “environment”) and exchange chemicals and energy (e.g., light), and behave accordingly, building up a stimulus-response dynamics. Current SCs are not alive, but recent progress allowed the construction of systems of ever-increasing complexity. **(B)** Schematic representation of a typical two-component signaling (TCS) system and the information flow. A transmembrane sensor S made of an input domain and kinase domain detects an outside signal X, resulting in the auto-phosphorylation of a conserved His residue in the kinase domain of S (at the expenses of ATP). Next, the sensor transfers the phosphoryl group to the conserved Asp residue on the receiver domain of a cognate response regulator R. In turn, the functional activity of the output domain is regulated, ultimately leading to an appropriate change in cellular physiology (typically, in gene expression pattern). **(C)** Distinct sensors (S1, S2) react differently to different inputs. From the viewpoint of fuzzy logic, sensors behave as fuzzy sets, which granulate the space defined by the chemical inputs. In this illustration, the input X belongs to the two molecular fuzzy sets at two different degrees (*x*_1_, *x*_2_) that are proportional to the interaction strength between the input X and the sensors. **(D)** Neural network-like line diagram representing possible cross-talks in artificial (engineered) TCS systems engrafted into SCs. In this diagram, a single input X can activate two sensors S1 and S2 (with different weights *x*_1_ and *x*_2_), which in turn activate two response regulators R1 and R2. The latter modify the gene expression pattern of genes G1 and G2. Dashed lines correspond to cross-talks, with weights *w*_12_, *w*_21_, *u*_12_, *u*_21_. More details in the text.

We will first introduce the motif of “phospho-neural networks” (Hellingwerf et al., 1995) and a plan for implanting such networks in SCs, calling for a specific design that would address both experimental feasibility, detailed modeling, and non-trivial behavior. We also suggest that the exploration and the interpretation of chemical networks’ dynamics, especially when they are based on macromolecular elements, is best pursued according to *fuzzy logic*. Finally, a short comment on the theoretical relevance of these approaches on the more general problem of embodying AI in the chemical domain will complete the paper.

## “Phospho-Neural Networks”

There have been several attempts to design chemical neural network (for a concise list, see (Blount et al., 2017)). Actually, most of them refer to hypothetical models—such as the Okamoto (Okamoto et al., 1987) and Ross (Hjelmfelt et al., 1991) approaches, while experimental results have been reported only recently, for example by exploring the DNA strand displacement strategy (Lakin and Stefanovic, 2016) or non-linear chemical systems communicating through UV-visible radiations (Gentili et al., 2017; Proskurkin et al., 2020) or reservoir computing (Nakajima and Fischer, 2021). The field of biochemical systems with neural network features is instead richer of examples, and has inspired several modeling studies (Fernando et al., 2009). Here we will focus on the potential neural network-like properties of signal transduction machineries in bacteria, and in particular of two-component signaling (TCS) systems (Figure 1B). We have been inspired by an enlightening and lucid report published some decades ago by Hellingwerf and collaborators (Hellingwerf et al., 1995), who also dubbed these networks as “phospho-neural networks”.

Indeed, the phosphorylation pathways that determine signal transduction and intracellular response in bacteria resemble a system of elements that are interconnected as neural networks. This can happen because, although TCS systems typically function in specific, parallel way, these information “channels” are far from being insulated, and information can cross-flow among them, giving rise to convergent or divergent branched pathways. While certain branched pathways are obligate in some cases (e.g., *Escherichia coli* chemotaxis systems, *Vibrio harveyi* quorum sensing signaling), being actually mandatory for a correct signalling (Agrawal et al., 2016), unwanted cross-talk can also happen (considered as noise), and cells evolved physiological mechanisms to prevent it.

However, the very tendency of cross-talking among these signalling channels implies the possible use of TCS sets as neural network—thus rising interest toward their potential use in SCs. The plan, thus, becomes the construction of intra-SC chemical neural networks, of minimal complexity, based on cross-talking TCS. Because bottom-up SCs have the advantage of well-defined chemical compositions, it is conceivable that conditions can be found in order to avoid cross-talk reducing processes, typically occurring *in vivo*. *In vitro* experiments have shown, indeed, that cross-talk phosphorylation reactions of the TCS elements spontaneously occur, and differ in reaction rates and specificity; moreover, convergent and divergent paths exist in some cases (Ninfa et al., 1988; Yamamoto et al., 2005; Agrawal et al., 2015).

It is worth noting that typical operations carried out by artificial neural networks (those operating in the logical domain of computers), such as “(machine) learning”, will not be easily exported to chemical neural networks. Concepts as the thresholds and weights, well-known to software developers working with neural networks, become intermolecular forces, rates of reaction, binding affinity when translated into the chemical domain. Biological phospho-neural networks, literally, have been learning during evolution of the organism(s) they belong to. On the other hand, current knowledge about TCS systems and technical capabilities in molecular biology offers intriguing opportunities for their rewiring and reprogramming, so to generate somehow novel (engineered) networks.

## An Intriguing Around-The-Corner Scenario

Hellingwerf et al. (1995) referred to phospho-neural network in bacterial cells. Today we ask new questions, which have both practical and theoretical perspectives: is it possible to engraft chemical neural networks, e.g., phospho-neural networks based on TCS, in SCs? Can they be a tool for implementing, in the wetware domain of the “sciences of artificial”, a sort of minimal chemical perceptron (McCulloch and Pitts, 1943)? Would it constitute a relevant example/outcome in terms of chemical embodied AI?

We believe that phospho-neural networks are approachable within current SC technology, although it is not an easy target. Therefore, we will cautiously speak about it as an around-the-corner scenario, something that is still unavailable right now, but can become affordable in the next few years. We will engage, then, in a speculative discussion about possible realizations of such systems. Our goal is to raise interest toward intra-SC chemical neural networks as an option for the design of next-generation cognitive SCs.

### Two-Component Signaling Systems Engrafted Into Synthetic Cells

Is it possible to engraft TCS systems into SC, for neural network-like operations? TCS systems mediate bacteria response to a wide range of signals and stimuli, such as nutrients, cellular redox state, pH, light, temperature, dissolved gases, quorum signals, hormones, osmolarity, antibiotics, etc. (Laub and Goulian 2007; Stock et al., 2000). Each TCS system consists of three elements (i.e., the three neuron-like elements): a sensor (histidine kinase) (S), a response regulator (R), and a gene (G) (Figure 1B). The sensor, which is a transmembrane protein, detects an environmental stimulus by its *N*-terminal domain, triggering the (auto)phosphorylation of a histidine residue in its kinase domain at the expenses of cellular ATP. In turn, the phosphorylated sensor reacts with its cognate response regulator, transferring the phosphate group to an aspartate residue of the regulator (phosphotransfer reaction). The typical final step is the control of gene expression by the phosphorylated response regulator, or—more in general—the control of cell physiology. Such systems have received many attentions in recent years and many topologies (connectivities) are known. We are interested, for the moment, to show that a minimal neural network based on TCS systems can be implemented in SCs. At this aim, a possible design could be based either on 1) TCS sets that are *per se* branched (e.g., one-to-many and many-to-one systems), such as the sporulation phosphorelay of *Bacillus subtilis* and the *M. tuberculosis* hypoxia sensing (Agrawal et al., 2016), or 2) TCS sets designed *ad hoc*, by rewiring TCS or enhancing the cross-talk in otherwise orthogonal TCS systems.

Engineering connectivity in TCS systems is considered within the experimental reach. Recent reviews are the good starting point for an up-to-date discussion about the technical possibilities and strategies (Laub and Goulian, 2007; Capra and Laub, 2012). For example, the substrate specificity of S can be reprogrammed by mutating as few as three residues (Skerker et al., 2008), and similar achievements have been obtained by mutating response regulators (Capra et al., 2010; Bell et al., 2010). Examples based on directed evolution are available (Siryaporn et al., 2010). The use of chimeric molecules (Wang et al., 2013) is encouraged by the modular structure of response regulators. In addition, downstream information flow can be engineered by a proper swapping of the response regulator DNA-binding domains (Schmidle et al., 2018), or by constructing proper promoters-ORFs (Open Reading Frames) combination.

Promoting cross-talk is also possible as summarized by another recent review (Agrawal et al., 2016) proposing different mechanisms, based on S/S or R/R heteromerization, on non-cognate S/R phosphorylation, on coregulation of the same gene by two different R, or on introducing auxiliary proteins that favour cross-talk. Another possibility relies on the simultaneous presence of wild-type and mutant S (and/or R), in order to display different kinetic preference toward their cognate elements. As mentioned, knowledge about TCS has grown considerably since the Hellingwerf *et al.* (1995) report. The issue of kinetic preference of an S for its cognate R could be somehow tuned or compensated by an appropriate design (easily realized in SCs, much less *in vivo*), which relies on the altered concentration of the various components or on kinetic counteractions (compensations) based on downhill processes (e.g., gene transcription, playing with concentrations of DNA or RNA polymerases; or at the level of mRNA availability).

The goal of implementing a chemical neural network inside SCs somehow mirrors its function *in vivo*, i.e., the integration of different signals into a *gene expression pattern* in specific manner, via convergent and divergent signaling that characterizes the neural networks. This will be considered as the first desired outcome, but a far-looking goal will be instead aimed at self-regulatory dynamics, possibly resulting in compensatory/adaptive/plastic dynamics. This could be achieved if the products of gene expression are elements of the phospho-neural network itself or effectors that change its behavior (so to achieve a sort of closed causal loop).

As mentioned, implanting a chemical neural network in current SCs will be technically challenging. The frontier components, i.e., the sensors S are integral membrane proteins, and thus their embedment in the SC membrane is not at all trivial, as it must face several issues like the optimal lipid composition of SC membrane (in order to favour their functionality), as well as their orientation. Membrane proteins can be embedded in SC membranes by reconstitution. Previous reports show that sensors of TCS systems have been successfully reconstituted in liposomes. For example, MtrB is involved in the osmostress response of *Corynebacterium glutamicum* and it has been reconstituted in functional way, by employing liposomes made of *E. coli* phospholipids (Möker et al., 2007). Similarly, PhoQ Mg^2+^-sensor from *Salmonella typhimurium* has been reconstituted in liposomes (Sanowar et al., 2005)*.* CpxA, the sensor of an *E. coli* TCS that detects envelop perturbation and it is also involved in biofilm formation, has been reconstituted in *E. coli* phospholipid nanodiscs (Hörnschemeyer et al., 2016). In all cases, the functionality of TCS systems was positively assessed, carrying out the phosphorylation pathway *in vitro*. Therefore, current knowledge could be employed as a guidance to 1) select systems that have been proved to function in liposomes; 2) reason about the best strategy for practical implementation of somehow similar “promising” systems. Alternatively, it could be attempted the direct *in situ* synthesis-and-insertion strategy of membrane proteins in SC membrane, from inside—typical of bottom-up autopoietic constructions (Kuruma et al., 2009; Altamura et al., 2017; Amati et al., 2020). Combinations of these approaches should be taken into consideration, recalling, however, that either detergent-based reconstitution either ribosomal synthesis-and-insertion require a careful design of the lipid composition of SC membrane, that must fulfil some requirements (form stable vesicles, be compatible with encapsulation procedure, allow the correct protein folding, do not interfere with protein synthesis and other intra-SC processes). Finally, the *in situ* synthesis of sensors and response regulators that require post-translational modifications can represent a further obstacle.

Despite these potential difficulties, we believe that implanting “minimal” phospho-neural networks in SCs is experimentally accessible, and that such plan would correspond to a major advancement in the field because it significantly adds to SC technology, especially from the viewpoint of developing artificial cognitive systems. Moreover, it contributes to merging SB and AI in a novel and potentially fruitful manner.

### Chemical Fuzzy Neural Networks

To complete the discussion, let us make a further step in the direction of chemical embodiment, by commenting on how chemical neural network could be modeled. We reasoned that in order to keep into account the real chemical nature of the neural network elements, which are proteins and thus can display conformational diversity, one needs to move from dichotomic two-state (yes/no) logic, as specified by sentences like “the sensor (or the response regulator) is/is not activated”, to the continuum case (gray-scale), typical of *fuzzy logic*. Thus, we further expect that intra-SC chemical neural network could become a useful study case of fuzzy logic application to complex chemical systems, and consequently allowing accurate modeling, insightful conclusions, and the departure from idealized binary logic.

We have emphasized that S and R elements of two different TCS systems can be specific or cross-talk (Agrawal et al., 2016), but, by the same reasoning, it is also possible that a single input signal can activate (at different degree) two similar sensors (e.g., nitrate activate, in *E. coli*, the two sensors NarX and NarQ (Noriega et al., 2010)). The schemes shown in Figures 1C,D describes both specificity and cross-talk. Note that it represents just a simple example of the neural network-like architectures we propose for SCs.

Firstly, it should be specified how parameters that are typically employed in neural networks are related to well-known physicochemical constants. Referring to Figures 1C,D, for instance, the degrees of membership *x*_1_ and *x*_2_ are related to the association constants of the chemical inputs to the two sensor proteins, S_1_ and S_2_; the weight coefficients *w*_*ij*_ are related to the kinetic constants (*k*_cat_, *K*_M_) of the phosphorylation reactions; the weight coefficients *u*_*ij*_ are related to the association constants of the phosphorylated R to the DNA. Examples of neural networks that map to chemical reactions have been reported, based on fundamental chemical laws (e.g., mass action, Arrhenius law) (Ji and Deng, 2021). The neural network “activation functions”, which is often a key parameter for the properly functioning of neural networks, should be based on physicochemical laws when referring to neural networks in the chemical domain. It would refer, therefore, to hyperbolic or sigmoidal isothermal binding curves, or to Michaelis-Menten profiles. Smooth analog input-output relationships are the most appropriate functions for processing the infinite-valued fuzzy logic. On the other hand, steep sigmoid functions are adequate for processing discrete logics, as it is Boolean binary logic (Gentili, 2011). The necessity of realistic modeling of chemical neural networks generates a set of constraints with respect to their operations and performance. Thus, what a chemical neural network can (and cannot) do, when compared to artificial neural network, represents one of the open questions that need to be addressed in future.

A fuzzy logic perspective starts from the consideration that each sensor (both S_1_ and S_2_) exists, in general, as a collection of conformers (Kenakin, 2011). Such conformational pluri-states confer every sensor the power of being sensitive to more than one input. However, distinct sensors react differently to different inputs. From this point of view, each sensor kinase behaves as a *fuzzy set* (Gentili 2014, 2018). Figure 1C shows that S_1_ and S_2_, being two molecular fuzzy sets, granulate the space defined by the chemical inputs. Distinct chemical inputs belong to the two molecular fuzzy sets at two different degrees. As an example, the figure shows that the input, represented as a chemical species X, belongs to both S_1_ and S_2_, but with two distinct degrees, which are *x*_1_ and *x*_2_. Each degree of membership *x*_*i*_ (*i* = 1 or 2) is proportional to the interaction strength between the input X and S_*i*_. The combination of the two-degree-of-membership values, *x*_1_ and *x*_2_, is transduced into a peculiar set of values for the neural network weights shown in Figure 1D. We have cross-talk whenever both degrees of membership, *x*_1_ and *x*_2_, are not null. Then, it might also be that:

$$w_{12}\neq 0\;\mathrm{and}/\mathrm{or}\;w_{21}\neq 0$$

$$u_{12}\neq 0\;\mathrm{and}/\mathrm{or}\;u_{21}\neq 0$$

The vector of network weight coefficients (*w*_11_, *w*_12_, *w*_21_, *w*_22_, *u*_11_, *u*_12_, *u*_21_, *u*_22_) is a function of the degrees of membership of the input to the two molecular fuzzy sets, which are the sensor proteins (i.e., S_1_ and S_2_):

$$\left(w_{11},\;w_{12},\;w_{21},\;w_{22},u_{11},\;u_{12},\;u_{21},\;u_{22}\right)=f\left(x_{1},\;x_{2}\right)$$

Specificity occurs when either

$$\left(x_{2}\;=\;0\right)\;\wedge \;\left(w_{12}\;=\;0\right)\;\wedge \;\left(u_{12}\;=\;0\right)$$

or when

$$\left(x_{1}\;=\;0\right)\;\wedge \;\left(w_{21}\;=\;0\right)\;\wedge \;\left(u_{21}\;=\;0\right)$$

It is worthwhile noticing that even the response regulators R and the genes G exist as collections of conformers. Therefore, they can exhibit an ensemble of reactivities, one for each conformer. It means that even R and G are fuzzy sets, and the network weight coefficients, *w*_*ij*_ and *u*_*ij*_ (*i*, *j* = 1 or 2), can assume—potentially—as many values as the number of reactive conformers. Clearly, the TCS systems are examples of chemical fuzzy neural networks. The possibility of processing fuzzy logic at the molecular level will simulate, in minimal way, some elementary yet fundamental features of biological intelligence (common to all organisms, from bacteria to human) to SCs such as that of making decisions in an environment of uncertainty, partiality and relativity of truth (Zadeh, 1973; Gentili, 2021) and that of recognizing variable patterns. In other words, SCs will become able to face complex scenarios and quickly adapt to an ever-changing environment.

## Concluding Remarks

The “performances” of chemical neural networks, especially when referred to minimal ones, cannot be compared to the very complex dynamics of artificial neural networks developed in the software domain, i.e., those on which the bright success of machine learning is based. Implications and relevance, instead, refer to the potential development of minimal cognitive SCs. In the wetware domain of the “sciences of the artificial”, indeed, it is possible to design and implement operations and circular dynamics that are instead unavailable/unproductive in the hardware and software domains. In particular, because molecules can act at the same time as operators (catalysts) and operands (substrates), chemistry blurs the traditional distinction between program and data, allowing self-modifying machines following a (closed) circular organization—the one that characterizes all living systems, from bacteria to humans. Although SCs with or without chemical neural networks do not realize such type of “closure”, reaching intermediate milestones such as those depicted in this article still adds to current knowledge, due to the “organizational relevance” of such artificial constructs (Damiano et al., 2011; Damiano and Stano, 2020).
